# Heart2Heart: a digital peer support programme for people with heart disease: protocol for a community-based, investigator-blinded randomised controlled trial conducted in Australia

**DOI:** 10.1136/bmjopen-2024-088740

**Published:** 2025-02-13

**Authors:** Julie Redfern, Emily Li, Andrew Maiorana, Robert Zecchin, Karice K Hyun, Wendan Shi, Joseph Weddell, Shuang Liang, Dion Candelaria, Tom Briffa, Adrian Bauman, Gemma Figtree, Maree L Hackett, Chi Kin Law, Qiang Tu, Richard Lindley, Robyn Gallagher

**Affiliations:** 1 Sydney Nursing School, University of Sydney, Sydney, New South Wales, Australia; 2 Institute for Evidence-Based Healthcare, Bond University, Gold Coast, Queensland, Australia; 3 Faculty of Medicine and Health, The University of Sydney, Sydney, New South Wales, Australia; 4 School of Physiotherapy and Exercise Science, Curtin University—Perth City Campus, Perth, Western Australia, Australia; 5 Cardiac Rehabilitation Services, Westmead Hospital, Westmead, New South Wales, Australia; 6 School of Health Sciences, University of Sydney, Camperdown, New South Wales, Australia; 7 The University of Sydney, Sydney, New South Wales, Australia; 8 Nursing, The University of Sydney, Sydney, New South Wales, Australia; 9 School of Population Health, University of Western Australia, Crawley, Western Australia, Australia; 10 School of Public Health, University of Sydney, Sydney, New South Wales, Australia; 11 Kolling Institute of Medical Research, Saint Leonards, New South Wales, Australia; 12 Faculty of Medicine, University of New South Wales, George Institute for Global Health, Camperdown, New South Wales, Australia; 13 Faculty of Health and Wellbeing, University of Central Lancashire, Preston, UK; 14 Faculty of Medicine and Health, The University of Sydney, Westmead, New South Wales, Australia; 15 Medicine, University of Sydney, Westmead, New South Wales, Australia

**Keywords:** Cardiovascular Disease, Coronary heart disease, Social Interaction, eHealth, REHABILITATION MEDICINE, Preventive Health Services

## Abstract

**ABSTRACT:**

**Introduction:**

Cardiac rehabilitation is known to reduce morbidity and improve quality of life in people living with heart disease, however, adherence, access and completion of these programmes is suboptimal. Peer support may offer an opportunity to close this service gap. The aim of the study is to determine whether the effectiveness of a digital peer support programme for people living with heart disease is effective in improving social connectedness, clinical and patient-reported outcomes and experience measures.

**Methods and analysis:**

Heart2Heart is a community-based randomised controlled trial with 6 months follow-up for the primary outcome and 6 and 12 months for secondary outcomes. Approximately 752 adults with a diagnosis of heart disease in the past 12 months will be recruited from the general community and Australian cardiac rehabilitation programmes. Control group will participate in usual care, while intervention group will have access to a 6 months intervention that enables peer support via an interactive mobile application, in addition to usual care. The intervention includes online discussion groups, access to resources and facilitated conversations with health professionals. Primary outcome is social connectedness at 6 months follow-up. Secondary outcomes (6 and 12 months) will be all-cause/cardiovascular disease hospital admissions, all-cause mortality, lifestyle (sufficiently physically active, not smoking, sufficient fruit and vegetable consumption), proportion taking prescribed medications and health service utilisation (medical appointments, cardiac rehabilitation, participation in any other in-person peer support activities). Patient-reported outcome and experience measures including self-efficacy, quality of life, satisfaction and programme engagement will be analysed at 6 months. Process measures will include application analytics, barriers and facilitators to engagement with the intervention from participant’s perspective. An intention-to-treat analysis will be used.

**Ethics and dissemination:**

Ethical clearance was obtained from Western Sydney Local Health District Ethics Committee. Heart2Heart has potential to improve social connectedness and provide a valuable addition to traditional cardiac rehabilitation.

**Trial registration number:**

ACTRN12624000386538.

**Strengths and limitations of this study**

- Heart2Heart is a community-based randomised controlled trial with 6 months follow-up for the primary outcome and 6 and 12 months for secondary outcomes.
- Heart2Heart will determine effectiveness of a digital peer support programme for people with heart disease on social connectedness based on a validated scale.
- Self-reported physical activity data will be objectively validated (accelerometers) in a 10% subset of participants.
- Barriers and enablers to delivery of and engagement with the digital peer support programme will be evaluated via a series of process measures.
- Intention-to-treat analysis will be conducted by a statistician blinded to group allocation.

## Introduction

 Cardiovascular diseases account for approximately one-third of all deaths globally.^1^ Coronary heart disease (CHD) is the leading cause of death attributable to CVD.^2^ Importantly, of the millions of people who experience acute coronary events each year, around a third occur in people who have prior CHD, and are largely preventable.^3^ Recent data from the SNAPSHOT Acute Coronary Syndrome (ACS) follow-up study found that almost 20% of people admitted to hospital with ACS die within 3 years of discharge and 40% experience another cardiovascular disease (CVD) hospitalisation in the same period.^4^ The most common structured preventive programme following an acute coronary event is cardiac rehabilitation; generally a time-limited outpatient group-based programme designed to deliver support, exercise and education sessions, although in recent years, digital modalities have emerged.^5 6^

Cardiac rehabilitation has been found to reduce new clinical events and improve quality of life and prognosis in people diagnosed with CHD.^7^ Participation in a structured cardiac rehabilitation programme is recommended in leading international guidelines.^8^,^10^ However, cardiac rehabilitation, referral, access and completion remain suboptimal.^11^ Reasons are well documented and include transportation impediments, work and/or social commitments and lack of perceived need.^12^ Furthermore, priority populations including those from culturally or linguistically diverse or low socioeconomic backgrounds are less likely to attend.^12^ Literature also suggests that women are less likely to be referred, to participate and complete cardiac rehabilitation programmes.^13^ At the same time, there are increasing numbers of people surviving initial acute coronary events meaning capacity for efficient and evidence-based support is increasing.^6^ Therefore, improving preventive care options and providing better support to people who both do, and do not, access or complete cardiac rehabilitation is a worldwide priority.^14^

Consumer-driven social networks and peer support programmes have great potential and value in healthcare due to their ability to promote ideas and information sharing between people and provide support in an economically viable manner. The concept of social connectedness has been defined as the ‘degree to which people have and perceive a desired number, quality, and diversity of relationships that create a sense of belonging, and being cared for, valued, and supported’.^15^ Consumer social networks are likely effective because they provide emotional support for behaviour change via social connectedness among members who possess experiential knowledge of similar situations and experiences.^15 16^ Meta-analyses have reported 50% increased likelihood of survival by people with stronger social connections^17^ and cohort studies have found that social isolation is associated with lower CVD screening^18^ and increased fatal first CVD events.^19^ Furthermore, randomised control trials and qualitative research among people postcardiac surgery^20^ and with diabetes have demonstrated the potential value of in-person social support networks in terms of reduced anxiety, better knowledge and improved self-efficacy for adopting healthier choices.^21 22^ However, previous research relating to peer support interventions has largely focused on in-person delivery.^23^ Furthermore, a study among people with diabetes found that a peer support was cost-saving,^24^ and a randomised trial involving men recovering from coronary artery bypass surgery found that the peer support intervention was associated with less health service utilisation; however, the authors highlight that further research in this area is needed.^25^

Advances in technology and affordable mobile phone technology offer an opportunity to maximise effectiveness and reach of preventive healthcare, both primary and secondary. Importantly, evidence suggests that digital health interventions should be consumer-focussed, meaningful and supportive for people who use them.^26^,^28^ A variety of studies have provided evidence that digital strategies have potential to improve access and outcomes while also being cost-effective^29^ and engaging for people with heart disease.^26^ Combining digital advancement and social connection via peer support offers a potentially innovative and scalable approach, but robust research with heart disease survivors is needed. Therefore, the aim of this study is to determine whether a community-based peer support mobile application for people living with heart disease improves social connectedness, hospitalisation, mortality, cardiovascular risk profile (including behavioural, biomarkers and medication persistence) and patient-reported measures. Also, the study aims to understand the perspectives of people with heart disease, clinicians and other stakeholder organisations in terms of barriers and enablers to engagement with peer support. We hypothesise that among people with heart disease, those randomised to a digital peer support programme will experience better social connectedness, health and patient-reported measures at 6 months follow-up than those randomised to usual care.

## Methods and analysis

### Study design

**Figure 1:**
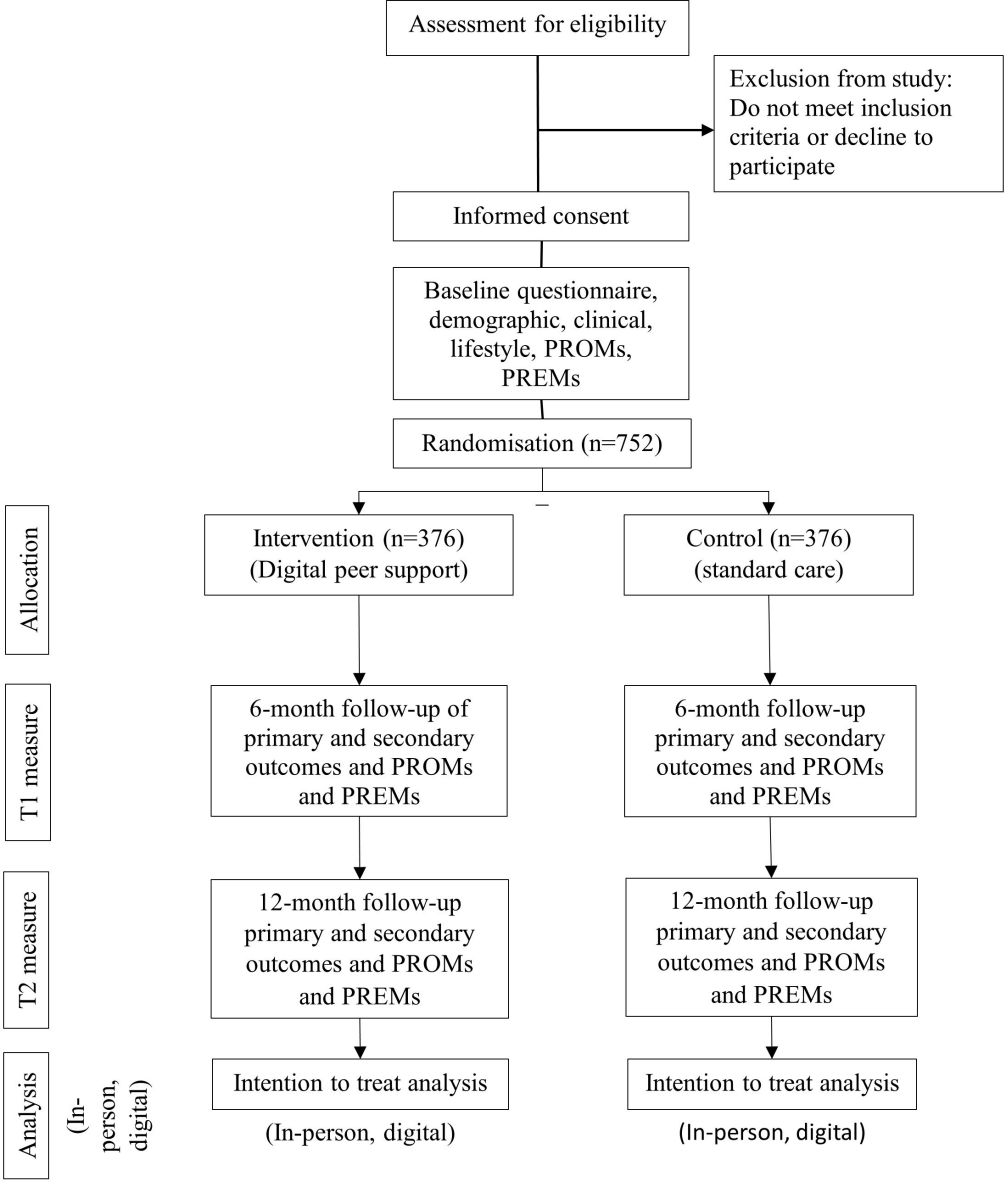
Study flow diagram. CONSORT, Consolidated Standards of Reporting Trials; PREMs, patient-reported experience measures; PROMs, patient-reported outcome measures.

Heart2Heart is a community-based, single-blind, randomised controlled trial (figure 1) with 6 and 12 month follow-up. The study will take place in three Australian states; New South Wales, Victoria and Western Australia from June 2024 to December 2025 (sponsor is University of Sydney). The protocol is reported according to the Standard Protocol Items: Recommendations for Interventional Trials (SPIRIT) Statement^30^ and intervention using the Template for Intervention Description and Replication (TIDieR).^31^

### Participants and recruitment

Inclusion criteria are people (1) aged at least 18 years; (2) with a confirmed diagnosis of heart disease, coronary revascularisation procedure or greater than 50% stenosis on coronary angiogram within the prior 12 months and (3) own a mobile device. Exclusion criteria are people (1) with major neurocognitive diagnoses; (2) who are not willing/able to give informed consent and (3) are unable to read or speak English.

Three recruitment strategies will be used to allow varied identification of potential participants via peer support associations, cardiac rehabilitation programmes and direct to consumers. The strategies are as follows: (1) consumer-focussed stakeholder organisations will email members with information about the study and if interested, potential participants can contact the research team for screening and to explore interest in participation; (2) the research team will contact community peer support programmes and promote the study via social media to introduce the trial to explore potential eligibility and interest of eligible people in participating and (3) cardiac rehabilitation coordinators in participating states will identify potential participants from their programmes over the previous 6 months. Research staff will then contact them in-person, via telephone or email to screen for eligibility and explore potential willingness to participate in the study. For all prospective participants, a research staff member will seek consent (online supplemental file 2) (either on paper or e-consent or directly via the mobile application) prior to baseline data collection and randomisation. Taken together, the collective cardiac rehabilitation programmes have approximately 1000 eligible people, the Australian stakeholder organisations have a membership base of over 1000 and the community peer support programmes include an online group (MyHeartMyLife) with approximately 3800 members.

### Randomisation and blinding

After informed consent, participants will be randomised and directed electronically to the online data collection survey. Randomisation will be stratified by regional/rural and urban areas and by those recruited from in-person peer support networks versus not (50:50). An independent statistician will generate the randomisation schedule (then upload into the study Research Electronic Data Capture, REDCap) with allocation sequence concealment strategies implemented. This is single-blind study where investigators, statisticians and research assistants collecting data will be blinded to group allocation; however, participants will know if they are allocated to the digital peer support intervention.

### Intervention

#### Control group

Participants allocated to the control group will continue to receive standard/usual care as determined by their treating healthcare professionals. That is participation in the study will not preclude them from their usual care. They will not have access to the digital peer support app because it is not publicly available.

#### Peer support intervention

Participants will have access to a 6 months mobile application (digital) peer support programme. The intervention aims to enable peer support among people with lived experience of heart disease via an online community forum. Details of the intervention are provided in table 1 based on the TIDieR checklist.^31^ On commencing the intervention period, participants will download the free application and register their details. Participants will then be able to access information and resources, select different discussion groups (general, diet and exercise, thinking and memory) based on topics of interest and interact with each other (table 1). All participants will have access to all Discussion Groups and will not be matched into pairs but form a large group. Registered health providers will be available to moderate the community topic discussions. Participants will also be provided with the Heart2Heart Peer Support Guide (Portable Document Format, PDF) via the mobile application in the resources tab. The Guide outlines the ground rules for participating in peer support sessions. Participants will also be asked to review the University of Sydney Privacy Policy, which outlines how their personal information will be used and protected.

**Table 1:** Overview of Heart2Heart intervention*

What	Access to a digital peer support app with four components; Discussion Groups with other people with lived experience of heart disease. The app has three primary discussion groups where participants can go online and introduce themselves and share their (1) general experiences relating to their heart disease journey and care, (2) exercise and diet and (3) memory and thinking. Participants will be able to freely move in and out of these discussion groups depending on their own preference. Ask an expert’ sessions that mimic those commonly available at the in-person peer support group. These sessions will be scheduled monthly for 30 min and include sharing of advice by multidisciplinary clinicians (registered with national relevant national authorities) such as nurses, allied health providers, dietitians and pharmacists. Participants will also be able to ask questions about topics such as exercise and diet, medicine and cognition. Resources are available via virtual folders in the app. Resources include videos and ‘fact sheets’ developed by the National Heart Foundation of Australia, peer support guidelines, the privacy policy and study documents. Fact sheets include resources relating to heart disease risk factors, medications and cardiac rehabilitation. The peer support guidelines cover aspects related to being respectful, inclusive and using appropriate language, promoting a supportive and welcoming community, encouraging a safe space, showing respect and empathy, using language carefully, listening to others a safe space where individuals feel comfortable sharing their stories and challenges.
Who	As a peer support intervention, the intervention is largely driven by conversations and input of other participants who also have lived experience of heart disease. However, the research team will monitor daily for inappropriate content (such as aggressive or inappropriate language or behaviour) and to help facilitate positive and supportive online conversation although content will be driven by the participants themselves.
How	The intervention is delivered online via a stand-along app that is only available to people allocated to the intervention group of this study. It is freely available and has a password-protected login. There is no face-to-face component.
When	Once signed into the app, participants can login and use it as much or as little as they choose for 12 months. There are no limits to time spent using the app although the study will track usage frequency and time spent in the app.
Tailoring	There is no personalised tailoring within the intervention

* Based on the Template for Intervention, dDescriptor and rReplication (TIDieR).^31^

### Data collection and study outcomes

The data collection schedule and time points for each outcome are summarised in table 2. Data will be collected remotely and entered into a purpose-built online questionnaire using the REDCap software.

**Table 2:** Schedule of Heart2Heart study procedures

	Time point (months)		
Schedule of assessments	0	6	12
Eligibility screen	✓		
Informed consent	✓		
Randomisation/allocation	✓		
Primary outcome			
Social connection in the context of cardiovascular health	✓	✓	✓
Secondary outcomes			
Social connectedness—8-item Social Connectedness Scale^33^	✓	✓	✓
Proportion physically inactive—GPAQ score^34^	✓	✓	✓
Proportion physically inactive—METs accelerometer (10% of participants)	✓	✓	
Point abstinence from smoking—self-report	✓	✓	✓
Proportion of participants consuming alcohol one or more standard drinks per week—self report	✓	✓	✓
Proportion of participants meeting fruits and vegetable guidelines—self-report	✓	✓	✓
Proportion of days covered with guideline-recommended medications—Pharmaceutical Benefits Scheme administrative data	✓	✓	✓
All-cause and CVD hospitalisations—self-report		✓	✓
All-cause and CVD mortality—National Death Index		✓	✓
Health Service Utilisation (medical appointments, cardiac rehabilitation, peer support programmes)—Medicare Benefits Scheme administrative data and self-report	✓	✓	✓
PROMs and PREMs			
Self-efficacy—Self-Efficacy to Manage Chronic Disease Scale—six item^35^	✓	✓	✓
Quality of life—EQ5D-5L^36^	✓	✓	✓
Participant satisfaction/acceptability with peer support programme—purpose built survey	✓	✓	
Digital peer support usage and engagement—analytical app data (page visit, frequency of login, participation in discussion groups, etc) for intervention participants only		✓	

CVDcardiovascular diseaseEQ5D-5LEuroQol 5 dimensionGPAQ, Global Physical Activity QuestionnaireMETsmetabolic equivalentsPREMspatient-reported experience measuresPROMspatient-reported outcome measures

The primary outcome is the proportion of participants who feel socially connected at 6 months follow-up. The primary outcome was selected to reflect the primary intent of the intervention and as a measure of the functional aspects (accessibility and utilisation of support) of social connection are the focus rather than structural aspects (eg, household numbers).^32^ The primary outcome is a composite of self-reported affirmative responses to two items related to functional social connection in the context of a person’s heart health and have been adapted from a validated survey.^33^ The two items are (1) ‘When it comes to my heart health, I am able to connect with other people’ and (2) ‘When it comes to my heart health, I have a sense of togetherness with my peers’. Response options for each item are ‘definitely not’, ‘probably not’, ‘maybe’, ‘yes, probably’ and ‘yes definitely’. Participants who report ‘yes probably’ and ‘yes definitely’ will be counted to the primary outcome.

Secondary outcomes (table 2) are differences in; mean aggregate social connectedness scale^33^ score, hospital admissions (all-cause and CVD), mortality, lifestyle (physical inactivity based on Global Physical Activity Questionnaire,^34^ not smoking, sufficient fruit and vegetable consumption), proportion taking guideline-indicated medications and health service utilisation (medical appointments, cardiac rehabilitation, community-based peer support programmes) between the control and intervention groups at 6 and 12 months. Patient-reported outcome and experience measures (table 2) are self-efficacy (Self-Efficacy to Manage Chronic Disease Scale,^35^ quality of life EQ5D-5L,^36^ satisfaction and programme (peer support engagement at 6 months)). Process measures will include application analytics, fidelity of the intervention and barriers and enablers to engagement from the perspective of participants allocated to the intervention group.

To verify self-reported physical activity data, a random subset (minimum 10%) of participants (control and intervention), physical activity will be assessed over 24 hours per day for 7 days (prior to baseline randomisation for baseline data), using a GENEActiv V.1.2 wrist-worn sensor which collects continuous accelerometry data via bluetooth. The sensor does not provide feedback to the wearer (no screen), minimising the likelihood of participants’ physical activity being influenced by sensor information. Sensors will be sent to and from participants via mail in a reply-paid envelope, along with instructions on how and when to wear the activity sensor and a logbook to record date and times worn or removed. Once the sensor and logbook are returned, the research team will download data from the tracker in real-time for storage in the study database. These data will be analysed by comparing average minutes per day participant were at least moderately physically active and be the subject of a sensitivity analysis to provide verification of the self-reported physical activity data.

An evaluation of the fidelity and barriers and enablers to delivery of and engagement with the digital peer support programme; a series of process measures will also be collected. A mixed-method approach will be used with four sources: (1) quantitative data from the app analytical data logs related to consumer engagement, participation in virtual communities, frequencies of logins, time spent on the app, rate of opening of resources within the peer support app and content of posts and responses; (2) focus groups with a subset of participants (all in intervention invited then from those who agree we will recruit consecutively until thematic saturation is achieved) in the intervention group to understand satisfaction with digital peer support, potential utility and suggestions for improvement and (3) semistructured interviews with a proportion of cardiac rehabilitation coordinators (all those from cardiac rehabilitation programmes who recruited participants will be invited) and stakeholders from not-for-profit peer support organisations (online search of Australian peer support associations for heart disease) to explore, from a clinician perspective, the usefulness of peer support, challenges to delivery, resources needed and requirements for implementation if such a programme was to be made routinely available. Sample sizes for the focus groups and semistructured interviews will be based on thematic saturation; however, based on prior experience,^37^ we anticipate that we will require at least four focus groups (6–8 participants per group) and 10 interviews.

### Data storage and management

De-identified data will be stored in the University of Sydney’s Research Data Store which is password protected. Identifiable information such as names, contact details, date of birth and post code will be removed from the data and be replaced with a participant ID number. Identifiable information will be stored separately from the clinical data on a locked electronic file location, which is only accessible by the research team.

### Sample size and statistical analyses

A sample size of 752 (376 per group) will achieve over 80% power, allowing for 10% drop-out, to detect a difference in the proportion of participants that feel socially connected between control and intervention groups by 10% at 6 months follow-up where the baseline estimate is based on the British Regional Heart Study, where 27% of participants with pre-existing CVD were classified as having low social engagement.^38^ As reported in a recent scientific state from the American Heart Association, there is consistent evidence for a direct association between social isolation, loneliness and CHD and stroke mortality, but the relationship is complex with unmeasured confounders.^39^ Based on advice from expert clinicians in the area, it was determined that an effect of reduced social isolation for one in 10 people would be clinically meaningful and hence an effect size of 10%.

Analyses will be performed according to the intention-to-treat principle by a statistician blinded to group allocation. Baseline and outcome data will be presented as frequencies and proportions for categorical data and means and SD for normally distributed continuous outcomes or median and IQR intervals for skewed continuous outcomes. Outcomes will be compared between control and intervention groups using all available data with intervention and control groups compared at 6 and 12 months. Normally distributed continuous outcomes will be analysed using the analysis of covariance and categorical outcomes will be compared using a log-binomial regression model, adjusting for baseline value of the outcome variable. Prespecified subgroup analyses will be performed for (1) self-reported gender, (2) regional areas, (3) cardiac rehabilitation completion (at least 50% of sessions offered) versus not and (4) previous or current peer support attendance (has participated in an in-person or other digital peer support programme since January 2024) versus not. The subgroups will be compared by including an interaction term between the subgroups and the randomisation status to the main models as described above.

## Ethics and dissemination

Lead ethical approvals have been obtained from the Western Sydney Local Health District Human Research Ethics Committee (2023/ETH00318) together with relevant site-specific governance approvals and amendments. Informed e-consent will be obtained from all participants. All individual and site information will be deidentified when reporting data and results to protect the confidentiality of participants. Identifiable data will only be required for the purpose of conducting data linkage with administrative data. Identifiers will be removed once data linkage has been completed. All participant data will be coded with an individual participant identifying number which will be used on all study documents. All study-related documents and data will be stored for a period of 5 years after publication of study results as per standard storage policies. Any hardcopy materials will be stored in locked filling cabinets and later shredded at the end of the 5 year period. Only research personnel directly involved with this project will have access to participants’ data.

Study data will be disseminated in peer-reviewed journals, at scientific meetings, to any organisations interested in translating the programme (local government, consumer not-for-profit organisations) and in the media. In any publications or presentations, group results will be discussed and will not identify individual participants.

### Patient and public involvement

People with heart disease have been involved throughout this study and the GRIPP2-SF checklist has been used to guide the reporting in this protocol and will also be used for the eventual outcomes papers online supplemental file 1.^40^ This includes focus groups and workshops with 28 people (16 male and 12 female) with lived experience of heart disease recruited from local in-person peer support groups to provide feedback on preferred peer support (in-person and digital) intervention content and to review a digital peer support prototype. These people provided feedback via an online survey, in-person focus groups and online workshops. Feedback included input regarding look and feel of the intervention, content, usability and meaningfulness. People participating in in-person peer support groups also participated in focus groups and shared their experiences with peer support and provided advice about what would be helpful to include. Together, this input from consumers was instrumental in the intervention design and development phase of the study. Consumer-focussed not-for-profit organisations (including Heart Support Australia, HeartBeat Victoria and HerHeart) are involved in the study and associated recruitment. Throughout the study, we aim to seek input and feedback from people with lived experience, clinicians and stakeholder organisations as part of the process measures. There is also a focus on PROMs and PREMs in the evaluation of the intervention as we aim to ensure consumer involvement and input throughout.

## supplementary material

10.1136/bmjopen-2024-088740 online supplemental file 1

10.1136/bmjopen-2024-088740 online supplemental file 2
