# Supplementary material for: Heart2Heart: a digital peer support programme for people with heart disease: protocol for a community-based, investigator-blinded randomised controlled trial conducted in Australia
Source: BMJ Open. 2025 Feb 13;15(2):e088740. doi: 10.1136/bmjopen-2024-088740 (PMC11831262; doi:10.1136/bmjopen-2024-088740)
Supplement: online supplemental file 1 [file bmjopen-15-2-s001.pdf]

Section and topic	Item	Reported on page Number
1: Aim	Report the aim of PPI in the study	8, 15
2: Methods	Provide a clear description of the methods used for PPI in the study	13, 15, 16
3: Study results	Outcomes—Report the results of PPI in the study, including both positive and negative outcomes	n/a
4: Discussion and conclusions	Outcomes—Comment on the extent to which PPI influenced the study overall. Describe positive and negative effects	15
5: Reflections/critical perspective	Comment critically on the study, reflecting on the things that went well and those that did not, so others can learn from this experience	15, 16

PPI - patient and public involvement

Supplementary Material: GRIPP2 short form checklist for Heart2Heart study
