# Supplementary material for: Heart2Heart: a digital peer support programme for people with heart disease: protocol for a community-based, investigator-blinded randomised controlled trial conducted in Australia
Source: BMJ Open. 2025 Feb 13;15(2):e088740. doi: 10.1136/bmjopen-2024-088740 (PMC11831262; doi:10.1136/bmjopen-2024-088740)
Supplement: online supplemental file 2 [file bmjopen-15-2-s002.pdf]

Participant Information Sheet/Consent Form

Non-Interventional Study - *Adult providing own consent*

**Title**	Heart2Heart: Implementation of a digital peer support program for people with heart disease
**Short Title**	Heart2Heart: A digital peer support program
**Coordinating Principal Investigator/ Principal Investigator**	Prof Julie Redfern
**Location**	Australia

Part 1 What does my participation involve?

1 Introduction

You are invited to take part in this research project, Heart2Heart. This is because you have expressed your interest to participate in this research trial relating to peer support program for people with heart disease. The research project is aiming to evaluate whether implementing a community led peer support program for people living with heart disease is feasible and cost-effective, reduces cardiovascular risk factors, and improve clinical and health outcomes.

This Participant Information Sheet/Consent Form tells you about the research project. It explains the tests and research involved. Knowing what is involved will help you decide if you want to take part in the research.

Please read this information carefully. Ask questions about anything that you don't understand or want to know more about. Before deciding whether or not to take part, you might want to talk about it with a relative, friend or local doctor.

Participation in this research is voluntary. If you don't wish to take part, you don't have to. You will receive the best possible care whether or not you take part.

If you decide you want to take part in the research project, you will be asked to sign the consent section. By signing it you are telling us that you:

- Understand what you have read
- Consent to take part in the research project
- Consent to the research as described
- Consent to the use of your personal and health information as described.

You will be given a copy of this Participant Information and Consent Form to keep.

2 What is the purpose of this research?

The primary aim of the study is to evaluate if implementing a community led peer support program for people with heart disease is feasible and cost effective, reduce related risk factors, and improve clinical and health outcomes. The secondary aim is to evaluate any barriers and enablers to implementation, expand the existing peer support program, and establish a national peer-support

registry. At the end of this study, it is anticipated that you will increase the awareness of cardiac rehabilitation, improve outcomes and long-term recovery by empowering you and others to support one another through sharing lived experience. The peer support programs could also be delivered nationally for people with heart disease and have the potential to be expanded across health conditions.

Currently, cardiac rehabilitation services have been underutilised with low rates of referral, attendance, and completion. This project intends to expand the knowledge in how peer support programs have the potential to address service access and provide additive benefits to existing services for people with heart disease.

The project is being led by Professor Julie Redfern, based at the University of Sydney and Western Sydney Local Health District, and is funded by the Australian Government's Medical Research Future Fund (MRFF) and the NSW Office of Health and Medical Research.

3 What does participation in this research involve?

If you volunteer, you will be participating in a community-based randomised controlled trial which involves a rollout of the peer support program. All those who volunteer to participate will have access to the peer support intervention for 6 months that includes digital format for linking in with other people with heart disease to share lived experience. Therefore, everyone will have access to the intervention by the end of the research study.

The research trial will take place across Australia.

Screening for eligibility and consent: If identified through the local community or your local cardiac rehabilitation coordinator as potentially suitable for the study, a research assistant will ask you some simple questions to determine if you are eligible for the study. To be eligible, you need to be i) at least 18 years old, ii) reside in Australia, iii) have confirmed diagnosis of heart disease; and iv) own an active mobile device. Participants with severe heart failure (NYHA class III or IV), neurocognitive diagnoses, who are not willing or able to give informed consent will be excluded. This screening process will take approximately 5 minutes. If the screening form shows that you meet the requirements, research assistants will then explain the study in more detail and offer you an opportunity to ask any questions you may have about the study. If you are interested in volunteering, you will be asked to review this information sheet and consent form. If you agree to volunteer, you will then be asked to sign the informed consent form. E-consent will be sought for participants who select digital intervention only.

Randomisation and blinding: If you volunteer to participate, you will be randomly allocated to either continue your usual care or to participate in a peer support program delivered by a mobile device or the internet. That is, like a toss of a coin, half the volunteers will be allocated to each group. For the study this will be done by a computer.

If allocated to the intervention group: you will have access a 6-month digital peer support program that enables interaction with other people with heart disease. The digital intervention will be delivered through a mobile application. You will download the free app and register your details. You will then be able to access information and resources, select different online communities based on topic interests e.g. exercise, medications, getting back to normal etc., interact with each other within your communities, and provide support in pairs (with similar history) and the broader social network. Health providers will be available to provide moderation of cardiac communities based on topic discussions.

Data collection: If you provide informed consent, the research assistant will then collect some simple health information and ask you to complete a series of questions about your heart health, quality of life and your perspective on managing health.

Data will be collected by research assistants (online surveys) including at the start and again 6 and 12 months later follow-ups. Each of these will take approximately 15-20 minutes to complete. Throughout the study, we will:

- ask you questions related to your social connectedness, physical activity, dietary, smoking, alcohol consumption, medication, medicine adherence, any use of medical services etc.
- evaluate your attendance and engagement.
- A small group of people will also be asked to collect data from a tracker watch about their physical activity for 1 week. The research team will contact you if you would like to participate in this aspect of the study.
- have access to the digital peer support application (app) software data to calculate how frequently participants visit and engage with pages and functionality in the app. The research team will also collect data about your health care usage and medication prescriptions via Services Australia and also if you happen to pass away (Australian Institute for Health and Welfare).

Focus groups: Some participants may be invited to participate in a 60-minute group discussion to explore your experiences with the peer support intervention. This is an additional voluntary component of the study and is not a requirement of participation in the peer-support study itself. We anticipate holding about a series of focus groups but numbers will vary to allow us to ensure we seek a broad range of experiences. We will provide an online gift card to the value of $50 as a reimbursement for participation in the focus group discussions. Focus groups will be recorded and transcribed and deidentified information will be stored in a secure database and will only be used for the purposes of this research study. None of your information will be shared with a third people and any person not part of the research team.

Linking to your Medicare health data: As part of this study, with your consent, we would like to link your information and health responses to your Medicare Benefits Schedule (MBS) and Pharmaceutical Benefits Scheme (PBS). These data will be obtained via Services Australia and will enable the team to collect information about what medications you are taking and what type of Medicare rebateable health services you are accessing. This will help the research team understand what the cost-benefit of a peer support program might be. This is an optional aspect of the study and to volunteer for this component you will be asked to provide your full name with your Medicare number and sign an additional consent form. Participation, or not, in this component does not restrict your participation in other aspects of the study. If you provide consent for this aspect of the study, your contact details will be sent to Services Australia who will electronically match your details with your MBS and PBS data and then return a password-protected file to the research team. You will be asked to sign a separate consent form authorising the study to access your Services Australia information, see the separate Services Australia Participant Information Document and Participant Consent Form.

Services Australia is not involved in this research other than to provide the information that you have consented to the release of, should you decide to participate in this study. Services Australia has confirmed that this research and any associated documents have received approval from a Human Research Ethics Committee (HREC) that is registered with and operates within guidelines set out by the National Health and Medical Research Council (NHMRC).

Death data: As part of our study, we aim to collect information including whether participants are still living. To achieve this, we will need to access death data called National Death Index (NDI). These data will be obtained from Australian Institute of Health and Welfare (AIHW) and enable the team to collect information about the date of death and cause of death, if any. This information is important for understanding the long-term impact of the intervention on any improvement in death. If you provide consent for this aspect of the study, your personal information (name, date of birth, sex, address, postcode) will be sent to AIHW who will electronically link your details with your NDI, if any, and then return a password-protected file to the research team.

A peer support registry: During the second year of this project, we will establish a consumer peer support registry of trained survivors. All participants have the option to join the online registry managed by the Heart2Heart research team. The registry will enable access to support and training resources, networking opportunities and connection beyond the life of the project. Information

collected via the Heart2Heart app will also enable virtual on-boarding evaluation, communication, and quality improvement.

Monitoring of risks: All risks associated with this research will be monitored and reported in accordance with the NHMRC Guidance for Safety Monitoring and Reporting in Clinical Trials. The research team will discuss any issues at regular study meetings and will immediately escalate to the Steering Committee if they cannot be resolved. Further, the team will undergo training in risk management and take a preventive approach to identifying, managing, and minimising risks by close monitoring of any risks and reporting incidents, risks and concerns to management.

Bias: This research project has been designed to make sure the researchers interpret the results in a fair and appropriate way and avoids researchers or participants jumping to conclusions.

Additional costs and reimbursement: There are not specific costs associated with participating in this research project, nor will you be paid. However, you will be reimbursed for any of the following costs that you incur as a result of participating in this research project. Those who participate in a focus group will receive a $50 gift card for their time.

It is desirable that your local doctor be advised of your decision to participate in this research project. If you have a local doctor, we strongly recommend that you inform them of your participation in this research project.

4 Do I have to take part in this research project?

Participation in any research project is voluntary. If you do not wish to take part, you do not have to. If you decide to take part and later change your mind, you are free to withdraw from the project at any stage. If you do decide to take part, you will be given this Participant Information and Consent Form to sign and you will be given a copy to keep.

Your decision whether to take part or not to take part, or to take part and then withdraw, will not affect your routine treatment, your relationship with those treating you or your relationship with The University of Sydney.

5 What are the possible benefits of taking part?

We cannot guarantee or promise that you will receive any benefits from this research, however your involvement in community-led peer support interventions will be of possible benefits to the broader community through adding knowledge about peer support for people with heart disease. You may also receive support from your peers and a health provider and be empowered to support others through sharing information and lived experience.

6 What are the possible risks and disadvantages of taking part?

Although all participants will be reminded that all discussions are to remain confidential, there is a possibility that other people participating in the program may not comply. To minimise this, all participants will be reminded not to disclose personal information to others during the peer support sessions. All participants will also be provided with the Heart2Heart Peer Support Guide that outline the ground rules for participating in peer support sessions when they start. Health professionals (medical, nursing, allied health) will be in place as a moderator to ensure safe communications among participants. All participants will also be asked to review the University of Sydney Privacy Policy (https://www.sydney.edu.au/policies/showdoc.aspx?recnum=PDOC2011/81&RendNum=0). This policy outlines how your personal health information will be used and protected. We will also ensure that current government and University COVID-19 regulations are adhered to for your safety.

If you become upset or distressed as a result of your participation in the research, the research team will be able to arrange for counselling or other appropriate support. Any counselling or support will be provided by qualified staff who are not members of the research project team. This counselling will be provided free of charge.

7 What if I withdraw from this research project?

If you decide to withdraw from this research project, please notify a member of the research team before you withdraw. A member of the research team will inform you if there are any special requirements linked to withdrawing.

If you do withdraw your consent during the research project, the research team will not collect additional personal information from you, although personal information already collected will be retained to ensure that the results of the research project can be measured properly and to comply with law. You should be aware that data collected by the sponsor up to the time you withdraw will form part of the research project results. If you do not want them to do this, you must tell them before you join the research project.

8 What happens when the research project ends?

You have an option to receive feedback about the overall results of this study. You can tell us that you wish to receive feedback by ticking the relevant box on the consent form. This feedback will be in the form of a one-page lay summary. You will receive this feedback after the study is finished.

Part 2 How is the research project being conducted?

9 What will happen to information about me?

By signing the consent form, you consent to the research team collecting and using personal information about you for the research project. Any information obtained in connection with this research project that can identify you will remain confidential. Your information will only be used for the purpose of this research project and it will only be disclosed with your permission, except as required by law.

Information about you may be obtained from your health records held at this and other health services for the purpose of this research. By signing the consent form, you agree to the research team accessing health records if they are relevant to your participation in this research project.

Your health records and any information obtained relevant to the study during the research project are subject to inspection for the purpose of verifying the procedures and the data. This review may be done by the relevant authorities and authorised representatives of the Sponsor, The University of Sydney, the institution relevant to this Participant Information Sheet, The University of Sydney, or as required by law. By signing the Consent Form, you authorise release of, or access to, this confidential information to the relevant research personnel and regulatory authorities as noted above.

All information will be treated confidentially and stored securely accordingly to ethical, legal and government regulations. Any identifiable information that is collected about you in connection with this study will remain confidential, will only be used for the purpose of this research project and will only be disclosed with your permission, or except as required by law. Results of this research project will be published or presented in such a way that you cannot be identified. If you request, we will provide you with a summary of the study results at the end of the study. Information about your participation in this research project may be recorded in your health records.

In accordance with relevant Australian and/or NSW and WA privacy and other relevant laws, you have the right to request access to the information collected and stored by the research team about you. You also have the right to request that any information with which you disagree be corrected. Please contact the research team member named at the end of this document if you would like to access your information.

Any information obtained for the purpose of this research project that can identify you will be treated as confidential and securely stored. It will be disclosed only with your permission, or as required by law.

10 Complaints and compensation

If you suffer any injuries or complications as a result of this research project, you should contact the study team as soon as possible and you will be assisted with arranging appropriate medical treatment. If you are eligible for Medicare, you can receive any medical treatment required to treat the injury or complication, free of charge, as a public patient in any Australian public hospital.

11 Who is organising and funding the research?

This research project is being conducted by Ms Wendan Shi, Mr Joseph Weddell, Dr Emily Li and Prof Julie Redfern as the lead investigator and is funded by Medical Research Future Fund and NSW Health.

12 Who has reviewed the research project?

The project has been reviewed by Medical Research Future Fund and NSW Health. All research in Australia involving humans is reviewed by an independent group of people called a Human Research Ethics Committee (HREC). The ethical aspects of this research project have been approved by the HREC of WSLHD HREC. This project will be carried out according to the National Statement on Ethical Conduct in Human Research (2007). This statement has been developed to protect the interests of people who agree to participate in human research studies.

13 Further information and who to contact

The person you may need to contact will depend on the nature of your query.
If you want any further information concerning this project, you can contact the research team on the following:

Clinical contact person

Name	*Julie Redfern*
Position	*Primary Investigator*
Telephone	*9351 2000*
Email	*Julie.redfern@sydney.edu.au*

For matters relating to research at the site at which you are participating, the details of the local site complaints person are:

Complaints contact person

Position	
Telephone	
Email	

If you have any complaints about any aspect of the project, the way it is being conducted or any questions about being a research participant in general, then you may contact:

Reviewing HREC approving this research and HREC Executive Officer details

Reviewing HREC name	*WSLHD Human Research Ethics Committee*
Executive Officer	*Kellie Hansen*
Telephone	*02 8890 9007*
Email	*Wslhd-researchoffice@health.nsw.gov.au*

Site RESEARCH GOVERNANCE Office contact

Position	
Telephone	
Email	

Consent Form - *Adult providing own consent*

**Title**	Heart2Heart: Implementation of a digital peer support program for people with heart disease
**Short Title**	Heart2Heart: A digital peer support program
**Coordinating Principal Investigator/ Principal Investigator**	Prof Julie Redfern
**Location**	Australia

Declaration by Participant

I have read the Participant Information Sheet and the University of Sydney Privacy Policy, or someone has read it to me in a language that I understand.

I understand the purposes, procedures (including hospital admission data from my state government department) and risks of the research described in the project.

I have had an opportunity to ask questions and I am satisfied with the answers I have received.

I freely agree to participate in this research project as described and understand that I am free to withdraw at any time during the project without affecting my future health care.

I acknowledge that any regulatory authorities may have access to my medical records **specifically related** to this project to monitor the research in which I am agreeing to participate. However, I understand my identity will not be disclosed to anyone else or in publications or presentations.

I understand that I will be given a signed copy of this document to keep.

Name of Participant (please print) ______________________________

Signature ______________________ Date ______________________

I would like to receive feedback about the overall results of this study YES ☐ NO ☐

If you answered **YES**, please provide your email address: ______________________

____

______________________ ______________________

* Witness is not to be the investigator, a member of the study team or their delegate. In the event that an interpreter is used, the interpreter may not act as a witness to the consent process. Witness must be 18 years or older.

Declaration by Researcher†

I have given a verbal explanation of the research project, its procedures and risks and I believe that the participant has understood that explanation.

Name of Researcher† (please print) ______________________________

Signature ______________________ Date ______________________

† A senior member of the research team must provide the explanation of, and information concerning, the research project.

Note: All parties signing the consent section must date their own signature.

Form for Withdrawal of Participation - *Adult providing own consent*

**Title**	Heart2Heart: Implementation of a digital peer support program for people with heart disease
**Short Title**	Heart2Heart: A digital peer support program
**Coordinating Principal Investigator/ Principal Investigator**	Prof Julie Redfern
**Location**	Australia

Declaration by Participant

I wish to withdraw from participation in the above research project and understand that such withdrawal will not affect my routine treatment, my relationship with those treating me or my relationship with The University of Sydney.

Name of Participant (please print) ______________________________

Signature ______________________ Date ______________________

Declaration by Study Doctor/Senior Researcher†

I have given a verbal explanation of the implications of withdrawal from the research project and I believe that the participant has understood that explanation.

Name of Researcher† (please print) ______________________________

Signature ______________________ Date ______________________

† A senior member of the research team must provide the explanation of and information concerning withdrawal from the research project.

Note: All parties signing the consent section must date their own signature.
