# Supplementary material for: Heart2Heart: a digital peer support programme for people with heart disease: protocol for a community-based, investigator-blinded randomised controlled trial conducted in Australia
Source: BMJ Open. 2025 Feb 13;15(2):e088740. doi: 10.1136/bmjopen-2024-088740 (PMC11831262; doi:10.1136/bmjopen-2024-088740)
Supplement: Supplementary file 3 [file bmjopen-2024-088740.reviewer_comments.pdf]

PEER REVIEW HISTORY

BMJ Open publishes all reviews undertaken for accepted manuscripts. Reviewers are asked to complete a checklist review form and are provided with free text boxes to elaborate on their assessment. These free text comments are reproduced below.

ARTICLE DETAILS

Title (Provisional)

Heart2Heart: A digital Peer support program for people with heart disease: protocol for a community-based, investigator-blinded randomised controlled trial conducted in Australia

Authors

Redfern, Julie; Li, Emily; Maiorana, Andrew; Zecchin, Robert; Hyun, Karice K; Shi, Wendan; Weddell, Joseph; Liang, Shuang; Candelaria, Dion; Briffa, tom; Bauman, Adrian; Figtree, Gemma; Hackett, Maree L; Law, Chi Kin; Tu, Qiang; Lindley, Richard; Gallagher, Robyn

---

VERSION 1 - REVIEW

**Reviewer**	**1**
**Name**	**Lof, Marie**
**Affiliation**	**Karolinska Institutet, Medicine, Huddinge**
**Date**	**12-Jul-2024**
**COI**	**I have no competing interests.**

Thank you for the opportunity to review this study protocol. It covers an interesting and not well studied topic: development and evaluation of an evidence-based digital tool for improving social connectedness for people living with CHD. Overall, the study protocol is well written and include a comprehensive description of the planned study. However, some clarifications are required.

Title: I would suggest removing the word "implementation". This is a traditional two-arm randomised controlled trial evaluating a peer support program. It is not a implementation study as there is no previous evidence of this program as far as I understand. Along the same lines, if it was an implementation study, I would expect a more homogenous recruitment base (e.g., through a health care system) instead of three different recruitment strategies.

Line 80: Re-phrase "Australian-only" to "English-speaking" or a similar expression based on language.

Line 99: Are women a priority population? I would remove women or re-phrase.

Lines 154-164: Please provide a rationale for the different recruitment strategies. To me , these channels seem somewhat different. Why have these been selected?

Line 176: Please expand on the conditions of the control group.

Lines 181-188: The description of the intervention content is too vague. For instance, what does "different discussion groups" mean? How are they formed? What is "access information and resources"? Please also describe more in detail HOW the information provided and the groups will improve the primary outcome. How is the intervention content theoretically grounded?

Line 226: When will physical activity be measured at baseline? Before or after randomisation. Please elaborate and how this procedure may impact the main findings.

Lines 226-228: Information on how the sensor data will be processed is completely lacking. What are the major variables that will be derived? Also, details on data collection regarding sampling frequencies and so on are lacking.

Lines 209-210: why are the outcomes sufficient fruit and vegetable consumption chosen? Why not model this as a continuous variable instead?

Lines 234-238: The process evaluation which also includes focus groups with end-users as well as health care providers is a major strength, however, please provide some more information the sample sizes for these studies as well as describe how they will be recruited.

Lines 287-297: Please provide data on the numbers of participants for each group.

**Reviewer**	**2**
**Name**	**Parry, Monica**
**Affiliation**	**University of Toronto Lawrence S Bloomberg Faculty of Nursing**
**Date**	**22-Jul-2024**
**COI**	**None.**

BMJ Open: Heart2Heart: implementation of a peer support program for people with heart disease: protocol for a community-based, investigator-blinded randomised controlled trial

General Comments. The authors have submitted a manuscript entitled *Heart2Heart: implementation of a peer support program for people with heart disease: protocol for a community-based, investigatorblinded randomised controlled trial* for consideration to BMJ Open. Although the authors have addressed an important and timely topic, there are a few significant gaps that require attention prior to publication of this manuscript. First, there is no specific framework that guides this study/trial. The authors are interested in the barriers and facilitators to implementation so I would suggest that an implementation framework (e.g.,

Consolidated Framework for Implementation Research [CFIR] be considered. This would provide the authors with a more robust systematic assessment of potential barriers and facilitators to the Heart2Heart implementation. Second, there are very few intervention details provided. The Template for Intervention Description and Replication (TIDieR) checklist should be used to detail the intervention. What are the specific elements of the intervention? What specifically would participants be engaged with during the 6 months of the study/trial? No peer specifics are included (who they are, what expertise do they bring, how they are trained, what information will they provide, how will the authors ensure no medical advice is provided, how often will peers interact with participants and how will this be done, will peers and participants be matched, etc.). Each of these should be provided in sufficient detail to enhance study/trial replication. Third, is this manuscript being considered for rapid publication based on successful funding? If so, reviewer comments with specific rebuttal statements need to be included. Lastly, protocol papers should report planned dates of study/trial completion and I did not see these reported in the submitted manuscript.

Detailed Comments

Background Literature. The authors are interested in a 6-month digital peer support intervention to improve social connectedness, clinical (all-cause/CVD hospital admissions, all-cause mortality, smoking/physical activity/dietary behaviors, health service utilization [medical appointments, cardiac rehabilitation, other peer support activities]), and patient-reported outcomes (self-efficacy, quality of life) and experiences (program engagement and satisfaction) in 752 adults diagnosed with coronary heart disease within the past year. They are also interested in process outcome measures (barriers and facilitators) as noted in the previous paragraph. Little details are provided in the background on sex/gender differences in cardiac rehabilitation referral, intake, and completion rates. A comment is made that previous research on peer support interventions have focused on in-person delivery; a reference is needed for this statement. Is there evidence that peer support affects mortality, if so, this should be included in the background to support all-cause mortality as an outcome. There is evidence that peer support interventions can increase costs, yet no mention of this is included in the background. Is this (i.e., cost) also being measured as a health care utilization outcome variable?

Research Design and Methods. I actually this this is a hybrid implementation trial versus a RCT.
Investigators will be blinded yet no details are provided to ensure this is a single-blinded study/trial.
Will caregivers (i.e., invisible patients) be considered as potential participants for this intervention? What if potential participants do not own a mobile device, are they able to participate in this study/trial? Including information on feasibility would strengthen the manuscript. A fairly large sample is needed, yet no details are provided about the consumer-focused stakeholder organizations, how many peer support programs currently exist and what is their demographic, and how many potential participants are anticipated for recruitment from cardiac rehabilitation programs? Who are the registered health providers that will moderate the community topic discussions, how often will this be done, and what are the community topic discussions? How will peers be recruited and trained, matched, etc.? The primary outcome includes two items that ask about social connectedness and peer togetherness. How will the participant distinguish from the social support available through the digital app from the social support available throughout their day-to-day recovery (i.e.,

embedded supports from the digital app support)? What exactly will be tracked as a measure of engagement? How will BMJ Open: Heart2Heart: implementation of a peer support program for people with heart disease: protocol for a community-based, investigator-blinded randomised controlled trial
engagement success be defined? What are the active ingredients of the digital app? A random sub-set of participants will wear a wrist-worn sensor, why? I do not see this as an objective of the study/trial. Who is a member of the virtual community? What resources are available in the digital app? Semistructured interviews will be completed with a proportion of cardiac rehabilitation coordinators and stakeholders; please clarify reason, type of questions, targeted numbers, etc. In terms of sample size, please clarify why 10% (27% to 37%) was chosen, does this represent a MCID? Pre-specified subgroup analyses will be performed for gender, please define gender. What does previous or current peer support attendance versus not mean in terms of your sub-group analyses? In the ethics and dissemination section the authors indicate that all study/trial related documents and data will be stored for five years after publication of study/trial results, then indicate in the next sentence that any hardcopy materials will be stored and then shredded at the end of the study/trial period, please clarify what is meant by this second statement. People with heart disease have been involved throughout the study/trial, please include a GRIPP 2 checklist to details the extent of involvement/collaboration. Who were 'these people' in terms of numbers, males/females/others, etc. Who were the not-for-profit organization involved to date? I also noticed the PHQ-9 questionnaire was listed as a measure in the ethics submission yet not mentioned in this protocol paper. Is depression also an outcomes variable? There is some risk with peer support interventions in terms of providing medical advice, sending more patients/participants for assessment to their primary care providers/hospital emergency rooms. Would a data management team not be useful/helpful to track this information as the study/trial is implemented? Is there risk of contamination? That is, will participants assigned to the usual care group be able to access the intervention, will this be tracked during study/trial implementation?

Conclusion. Results and conclusions are usually not included with study/trial protocols.

VERSION 1 - AUTHOR RESPONSE

REVIEWER: 2

1. First, there is no specific framework that guides this study/trial. The authors are interested in the barriers and facilitators to implementation so I would suggest that an implementation framework (e.g., Consolidated Framework for Implementation Research [CFIR] be considered. This would provide the authors with a more robust systematic assessment of potential barriers and facilitators to the Heart2Heart implementation. Second, there are very few intervention details provided.

Response: Thank you for these suggestions. We apologise for any confusion. This is a standard RCT as per comments of Reviewer 2 and to date there is not specific evidence of effectiveness. Hence, we have not opted to use CFIR or similar. To avoid confusion we have updated language related to barriers and enablers to make it clearer this aspect is about evaluating delivery of and engagement with the intervention rather than implementation as

such (in title and throughout the revised manuscript). We agree that this will help inform future implementation studies if the RCT is positive. We have also now provided more specific details about the intervention as requested and guided by TIDieR as recommended in point #2 below.

2. **The Template for Intervention Description and Replication (TIDieR**) checklist should be used to detail the intervention. What are the specific elements of the intervention? What specifically would participants be engaged with during the 6 months of the study/trial? No peer specifics are included (who they are, what expertise do they bring, how they are trained, what information will they provide, how will the authors ensure no medical advice is provided, how often will peers interact with participants and how will this be done, will peers and participants be matched, etc.). Each of these should be provided in sufficient detail to enhance study/trial replication. Third, is this manuscript being considered for rapid publication based on successful funding? If so, reviewer comments with specific rebuttal statements need to be included. Lastly, protocol papers should report planned dates of study/trial completion and I did not see these reported in the submitted manuscript.

Response: As suggested the TIDieR checklist has now been completed and we agree this helps strengthen and provide clarity in relation to the Intervention. This has also been incorporated into the revised manuscript (Table 1) and the full checklist uploaded as a Supplementary Material. We have also provided more clarity in relation to the elements of the intervention that participants will engage with, peer training/skills, oversight from research team. To clarify, this is a large group in an online community with no matching. This manuscript is not being considered for rapid publication. Study dates have been included in the revised manuscript also as per Editor suggestion.

3. **Background Literature**. The authors are interested in a 6-month digital peer support intervention to improve social connectedness, clinical (all-cause/CVD hospital admissions, all-cause mortality, smoking/physical activity/dietary behaviors, health service utilization [medical appointments, cardiac rehabilitation, other peer support activities]), and patient-reported outcomes (self-efficacy, quality of life) and experiences (program engagement and satisfaction) in 752 adults diagnosed with coronary heart disease within the past year. They are also interested in process outcome measures (barriers and facilitators) as noted in the previous paragraph. Little details are provided in the background on sex/gender differences in cardiac rehabilitation referral, intake, and completion rates. A comment is made that previous research on peer support interventions have focused on in-person delivery; a reference is needed for this statement. Is there evidence that peer support affects mortality, if so, this should be included in the background to support all-cause mortality as an outcome. There is evidence that peer support interventions can increase costs, yet no mention of this is included in the background. Is this (i.e., cost) also being measured as a health care utilization outcome variable?

Response:

- We have added text to the revised Introduction in relation to sex/gender differences in cardiac rehabilitation referral, intake and completion rates as suggested (line 108-109).
- A reference has been provided in relation to the lack of research relating to digital peer support (line 130).
- We are unaware of any studies to date that have examined the effect of peer support on CVD mortality (we are happy to be corrected) and have included mortality as a secondary outcome for completeness and future availability of data that could be combined with other studies in future meta-analyses.

- We are unaware of any studies to date that have found peer support interventions for people with CVD increases cost (we are happy to be corrected) however, we are aware of a study that demonstrated cost saving of peer support among people with diabetes and do acknowledge there could be increased health service utilisation as a result of peer support. We have now highlighted this issue in the revised manuscript (lines 130-134). We currently do not have funds for a cost-effectiveness analysis but this is a good suggestion and we will aim to seek further funding to do so and agree service utilisation would be an important part of that.

4. **Research Design and Methods**. I actually this this is a hybrid implementation trial versus a RCT. Investigators will be blinded yet no details are provided to ensure this is a single-blinded study/trial. Will caregivers (i.e., invisible patients) be considered as potential participants for this intervention? What if potential participants do not own a mobile device, are they able to participate in this study/trial? Including information on feasibility would strengthen the manuscript. A fairly large sample is needed, yet no details are provided about the consumer-focused stakeholder organizations, how many peer support programs currently exist and what is their demographic, and how many potential participants are anticipated for recruitment from cardiac rehabilitation programs? Who are the registered health providers that will moderate the community topic discussions, how often will this be done, and what are the community topic discussions? How will peers be recruited and trained, matched, etc.? The primary outcome includes two items that ask about social connectedness and peer togetherness. How will the participant distinguish from the social support available through the digital app from the social support available throughout their day-to-day recovery (i.e., embedded supports from the digital app support)? What exactly will be tracked as a measure of engagement? How will engagement success be defined? What are the active ingredients of the digital app? A random sub-set of participants will wear a wrist-worn sensor, why? I do not see this as an objective of the study/trial. Who is a member of the virtual community? What resources are available in the digital app? Semi-structured interviews will be completed with a proportion of cardiac rehabilitation coordinators and stakeholders; please clarify reason, type of questions, targeted numbers, etc. In terms of sample size, please clarify why 10% (27% to 37%) was chosen, does this represent a MCID? Pre-specified sub-group analyses will be performed for gender, please define gender. What does previous or current peer support attendance versus not mean in terms of your sub-group analyses? In the ethics and dissemination section the authors indicate that all study/trial related documents and data will be stored for five years after publication of study/trial results, then indicate in the next sentence that any hardcopy materials will be stored and then shredded at the end of the study/trial period, please clarify what is meant by this second statement. People with heart disease have been involved throughout the study/trial, please include a GRIPP 2 checklist to details the extent of involvement/collaboration. Who were 'these people' in terms of numbers, males/females/others, etc. Who were the not-for-profit organization involved to date? I also noticed the PHQ-9 questionnaire was listed as a measure in the ethics submission yet not mentioned in this protocol paper. Is depression also an outcomes variable? There is some risk with peer support interventions in terms of providing medical advice, sending more patients/participants for assessment to their primary care providers/hospital emergency rooms. Would a data management team not be useful/helpful to track this information as the study/trial is implemented? Is there risk of contamination? That is, will participants assigned to the usual care group be able to access the intervention, will this be tracked during study/trial implementation?

Response:

- As per point #1 and Reviewer 2 this is an effectiveness trial as there is no compelling evidence for implementation at this stage and we have revised the manuscript accordingly.
- By single blind we refer to the fact that investigators and statisticians will be blinded to group allocation for the purpose of data collection and analysis but we are unable to blind participants as they will know if they have access to the digital peer support program. This has been clarified in the revised manuscript (lines 192-195).
- Caregivers (i.e., invisible patients) are not eligible to be participants as per inclusion criteria (i) stating participants must have a confirmed diagnosis heart disease (line 163).
- Potential participants must own a mobile device to be eligible to participate (line 165).
- Clarification has been provided in the revised manuscript in relation to feasibility (lines 181-185).
- The health providers are registered and this is now specified in the revised manuscript (Table 1).
- Participants will interact within the app in a single large group without specific matching or training and this is clarified in the revised manuscript (lines 210-211). In the resources section of the app there is guidelines on peer support available and the discussions will be overseen by the research team (Table 1).
- The primary outcome (social connectedness and peer togetherness) will measure whether our intervention improves these aspects, with randomisation ensuring our results can be attributed to the addition of the intervention to day-to-day recovery. Pre-specified sub-group analyses will be conducted in relation to cardiac rehabilitation completion versus not and previous or current peer support attendance (now defined in revised manuscript) versus not (lines 305-307).
- To track measures of engagement, we will have access to quantitative analytical data from the app including frequency of logins, page views, participation in virtual communities, time spent on the app, rate of opening of resources and content of posts and this has been clarified in the revised manuscript (lines 264-267). We have not specifically define engagement success as these measures are an indicator of barriers and enablers and levels of engagement that can be used to inform future development and studies. We will also be able to track whether engagement is sustained and conduct per protocol analyses excluding those who never engage.
- The active ingredients in the digital app are now summarised in Table 1 that is based on the TIDieR Checklist along with a summary of the types of resources that are available (Table 1).
- The random sub-set of participants wearing the accelerometers is to provide some cross-checking for the self-reported physical activity. Due to budget constraints and for feasibility reasons this was not possible for the whole sample. We agree this was not made specific in the original manuscript but have now included this in the revised version (lines 248).
- Anticipated sample size for the semi-structured interviews was also raised by Reviewer 1 and is now included in the revised manuscript and we have clarified the types of probes that will be explored (lines 276-279).
- In terms of sample size, we agree the 27-37% was confusing so this has been removed. The study is powered for a 10% effect size in relation to social connectedness.
- For the pre-specified sub-group analyses we will compare self-reported gender and this is now clarified in the revised manuscript (line 304).

- For study/trial related documents and data storage this is 5-years and the second sentence has been edited to clear this up so rather than "the study period" it now says the 5-year period" in the revised manuscript (line 322).
- In terms of involvement of people with heart disease, we have completed and uploaded the GRIPP2-SF Checklist as suggested and provided more detail about the people involved to date and stakeholder organisations in the revised manuscript and a statement that this checklist will also be used in the outcomes paper (lines 331-347).
- Neither PHQ-9 or depression are outcomes. Neither are listed in the manuscript, Table 1 or the ANZCTR. EQ-5D will be or QoL assessment and is in the manuscript, Table 1 and the ANZCTR. Ethics approval has been amended since this BMJ Open submission with the PHQ9 now removed.
- Participants in the control group will not be able to access the app (lines 200-201) as it is via registration only after randomisation and login information is only given to the intervention group and the app is not publicly available. Yes we will have health service utilisation data in relation to primary care and self-report for hospitalisations. This is a good point raised and also speaks to cost. We will compare health service utilisation in the control and intervention groups. We will not have access to this data until the conclusion of follow-up and hence this will not be tracked in real-time.

Conclusion. Results and conclusions are usually not included with study/trial protocols.
Response: Conclusion removed as suggested

REVIEWER: 1
Overall, the study protocol is well written and include a comprehensive description of the planned study. However, some clarifications are required.

Title: I would suggest removing the word "implementation". This is a traditional two-arm randomised controlled trial evaluating a peer support program. It is not a implementation study as there is no previous evidence of this program as far as I understand. Along the same lines, if it was an implementation study, I would expect a more homogenous recruitment base (e.g., through a health care system) instead of three different recruitment strategies.
Response: We agree this has caused confusion and have removed the term in the title and manuscript.

Line 80: Re-phrase "Australian-only" to "English-speaking" or a similar expression based on language.
Response: This has now been removed as a result of re-writing these dot points.

Line 99: Are women a priority population? I would remove women or re-phrase.
Response: Removed as suggested.

Lines 154-164: Please provide a rationale for the different recruitment strategies. To me , these channels seem somewhat different. Why have these been selected?
Response: Our intention is to recruit via in-person peer support groups and there are a number of associations that have newsletters and databases of potential participants. We also wanted to recruit people completing cardiac rehabilitation as this is a time when patients are often a little lost and unsure about future support. We also wanted to recruit direct from consumers. The 3 strategies help enhance generalisability and feasibility of the study. This is now clarified in the revised manuscript (lines 169-177).

Line 176: Please expand on the conditions of the control group.
Response: Expanded as requested (lines 198-201).

Lines 181-188: The description of the intervention content is too vague. For instance, what does "different discussion groups" mean? How are they formed? What is "access information and resources"? Please also describe more in detail HOW the information provided and the groups will improve the primary outcome. How is the intervention content theoretically grounded?
Response: We agree this was far too light. This has now been expanded considerably and was also raised by Reviewer #2. We have also used the TIDieR checklist to guide this reporting. See earlier responses to Reviewer #2.

Line 226: When will physical activity be measured at baseline? Before or after randomisation. Please elaborate and how this procedure may impact the main findings.
Response: The physical activity questionnaire and accelerometer (where) allocated data is collected prior to randomisation. By doing this before randomisation we are intending to reduce response-bias.

Lines 226-228: Information on how the sensor data will be processed is completely lacking. What are the major variables that will be derived? Also, details on data collection regarding sampling frequencies and so on are lacking.
Response: In the revised manuscript we have provided more detail about the sensor data (lines 248-260). These will be collected for 7 days using GENEActiv 1.2 trackers. These trackers provide no visual feedback for participants and can be worn for 24 hours per day. Prior to randomisation to control or intervention, the subset of participants allocated to this sub-group will be posted the accelerometer to be worn for the week prior to group allocation and then post it by via a reply-paid postage envelope. Once received by the research team the data is extracted and provide data on minutes per day the participant was partaking moderate physical activity. Analyses will then compare groups using ANOVA in terms of average minutes per day of moderate physical activity.

Lines 209-210: why are the outcomes sufficient fruit and vegetable consumption chosen? Why not model this as a continuous variable instead?
Response: We aimed for categorical to align with numbers of servings in guidelines, that is meeting guidelines for fruit and vegetables or not.

Lines 234-238: The process evaluation which also includes focus groups with end-users as well as health care providers is a major strength, however, please provide some more information the sample sizes for these studies as well as describe how they will be recruited.
Response: This is a good point and we apologise for the oversight. This will be dependent on thematic saturation but we have provided an estimate, based on experience, in the revised manuscript (lines 276-279).

Lines 287-297: Please provide data on the numbers of participants for each group.
Response: This is provided in the sample size section of the manuscript (line 289).

VERSION 2 - REVIEW

**Reviewer**	**1**
**Name**	**Lof, Marie**
**Affiliation**	**Karolinska Institutet, Medicine, Huddinge**
**Date**	**04-Jan-2025**
**COI**	

The authors have addressed all my comments. I am happy to see that it is now referred to as an effectiveness trial and not as implementation science.
